# SK4 channels modulate Ca^2+^ signalling and cell cycle progression in murine breast cancer

**DOI:** 10.1002/1878-0261.12087

**Published:** 2017-06-26

**Authors:** Friederike A. Steudel, Corinna J. Mohr, Benjamin Stegen, Hoang Y. Nguyen, Andrea Barnert, Marc Steinle, Sandra Beer‐Hammer, Pierre Koch, Wing‐Yee Lo, Werner Schroth, Reiner Hoppe, Hiltrud Brauch, Peter Ruth, Stephan M. Huber, Robert Lukowski

**Affiliations:** ^1^ Department of Pharmacology, Toxicology and Clinical Pharmacy Institute of Pharmacy University of Tuebingen Germany; ^2^ Dr. Margarete Fischer‐Bosch‐Institute of Clinical Pharmacology Stuttgart and University of Tuebingen Germany; ^3^ Department of Radiation Oncology University of Tuebingen Germany; ^4^ Department of Pharmacology and Experimental Therapy Institute of Experimental and Clinical Pharmacology and Toxicology University Hospital Tuebingen Germany; ^5^ Pharmaceutical and Medicinal Chemistry Institute of Pharmacy University of Tuebingen Germany; ^6^ German Cancer Consortium (DKTK) German Cancer Research Center (DKFZ) Heidelberg Germany

**Keywords:** breast cancer, Ca^2+^‐activated K^+^ channels of intermediate conductance (SK4, K_Ca_3.1, IK, *KCNN4*), epidermal growth factor receptor 2 (Her2/cNeu), mouse mammary tumour virus (MMTV), polyoma virus middle T‐antigen (PyMT)

## Abstract

Oncogenic signalling via Ca^2+^‐activated K^+^ channels of intermediate conductance (SK4, also known as K_Ca_3.1 or IK) has been implicated in different cancer entities including breast cancer. Yet, the role of endogenous SK4 channels for tumorigenesis is unclear. Herein, we generated SK4‐negative tumours by crossing SK4‐deficient (SK4 KO) mice to the polyoma middle T‐antigen (PyMT) and epidermal growth factor receptor 2 (cNeu) breast cancer models in which oncogene expression is driven by the retroviral promoter MMTV. Survival parameters and tumour progression were studied in cancer‐prone SK4 KO in comparison with wild‐type (WT) mice and in a syngeneic orthotopic mouse model following transplantation of SK4‐negative or WT tumour cells. SK4 activity was modulated by genetic or pharmacological means using the SK4 inhibitor TRAM‐34 in order to establish the role of breast tumour SK4 for cell growth, electrophysiological signalling, and [Ca^2+^]_i_ oscillations. Ablation of SK4 and TRAM‐34 treatment reduced the SK4‐generated current fraction, growth factor‐dependent Ca^2+^ entry, cell cycle progression and the proliferation rate of MMTV‐PyMT tumour cells. *In vivo*, PyMT oncogene‐driven tumorigenesis was only marginally affected by the global lack of SK4, whereas tumour progression was significantly delayed after orthotopic implantation of MMTV‐PyMT SK4 KO breast tumour cells. However, overall survival and progression‐free survival time in the MMTV‐cNeu mouse model were significantly extended in the absence of SK4. Collectively, our data from murine breast cancer models indicate that SK4 activity is crucial for cell cycle control. Thus, the modulation of this channel should be further investigated towards a potential improvement of existing antitumour strategies in human breast cancer.

## 1 Introduction

Breast cancer is the most common malignant disease among women and a major cause of cancer‐related mortality in the developed world (Senkus *et al*., 2015; Siegel *et al*., 2016). Breast cancer heterogeneity resulting in variable therapy response and survival emphasizes the need for novel diagnostic, prognostic and predictive markers regulating different aspects of tumour cell behaviour in order to improve disease management (Lastraioli *et al*., 2015).

Members of the Ca^2+^‐activated K^+^ channel (K_Ca_) family, that is BK (also known as K_Ca_1.1, Maxi‐K or Slo1) and SK4 (also known as K_Ca_3.1 or IK), have been implicated in the progression of malignant diseases including but not limited to gliomas (D'Alessandro *et al*., 2013; Liu *et al*., 2002; Turner *et al*., 2014), clear cell renal cell carcinoma (Rabjerg *et al*., 2015) and breast cancer (Haren *et al*., 2010; Oeggerli *et al*., 2012; Thurber *et al*., 2017). Aberrant K^+^ efflux via oncogenic K_Ca_ channels may affect multiple parameters in the breast tumour cell such as membrane potential, cytosolic Ca^2+^ ([Ca^2+^]_i_), pH and cell volume (Huang and Jan, 2014; Huber, 2013). Current knowledge suggests that BK (Lallet‐Daher *et al*., 2009; Parihar *et al*., 2003; Stegen *et al*., 2015) and SK4 (Ouadid‐Ahidouch *et al*., 2004; Parihar *et al*., 2003; Steinle *et al*., 2011) activities are required for malignant growth of several tumour‐derived cell lines and of xenografts in immunocompromised mice, highlighting a general role of K_Ca_ channels for cell cycle‐specific functions (Huang and Jan, 2014; Pardo and Stuhmer, 2014). Expression of SK4 and BK in cancer cells follows a cell cycle‐dependent mode (Ouadid‐Ahidouch *et al*., 2004; Pardo *et al*., 1998) and the mitogen‐dependent regulation of K_Ca_ activity supports a role for both channels in malignant (Faouzi *et al*., 2010; Lallet‐Daher *et al*., 2009; Wang *et al*., 2007a) and nonmalignant cell proliferation (Grgic *et al*., 2005; Khanna *et al*., 1999; Toyama *et al*., 2008; Yu *et al*., 2013). By inducing a more negative membrane voltage, activation of K^+^ channels provides a driving force for Ca^2+^ influx into the nonexcitable tumour cell. In addition, by modifying the membrane potential, K^+^ channels regulate voltage‐dependent activity and/or conductivity of Ca^2+^ entry pathways (Huang and Jan, 2014; Huber, 2013). Because Ca^2+^ is an ubiquitous intracellular messenger relevant for cell survival, apoptosis and proliferation (Azimi *et al*., 2014; Monteith *et al*., 2007), it was proposed that breast tumour K_Ca_ channels promote pro‐oncogenic functions at least in part by Ca^2+^‐dependent signalling pathways (Kunzelmann, 2005; Ouadid‐Ahidouch and Ahidouch, 2013). Oscillations of [Ca^2+^]_i_ have been observed during exit from G_1_, during S phase, and at the end of M phase (Parkash and Asotra, 2010; Santella *et al*., 2005). A hyperpolarization of the breast tumour cell membrane reportedly resulted in elevated [Ca^2+^]_i_ (Wang *et al*., 1998), which in turn may activate K_Ca_ to control spatiotemporal Ca^2+^ dynamics. Accordingly, the SK4 inhibitors TRAM‐34 and clotrimazole reduced Ca^2+^ entry and cell cycle progression as shown for colon and endometrium cancer cells (Lai *et al*., 2011; Wang *et al*., 2007b). In the metastatic breast cancer cell line MDA‐MB‐231, suppression of SK4 channels with TRAM‐34 or siRNA inhibits both cell proliferation and migration and induces apoptosis (Zhang *et al*., 2016). Moreover, overexpression of SK4 in MDA‐MB‐231 cells increased primary tumour growth and metastasis (Thurber *et al*., 2017), whereas TRAM‐34 at concentrations known to inhibit plasma membrane SK4 channels stimulates the proliferation of MCF‐7 cells presumably by a mechanism that involves oestrogen receptors (Roy *et al*., 2010). Besides direct effects on the tumour cell, TRAM‐34‐sensitive SK4 channels decreased the ability of natural killer cells to suppress tumour grafts in the NOD‐SCID mouse model (Koshy *et al*., 2013). Obviously*,* further studies are needed to clarify the pharmacological properties of SK4 channels in nontumour/tumour cells and in breast cancer. Due to reported nonspecific effects of TRAM‐34 and other compounds modulating SK4 (Agarwal *et al*., 2013; Wulff *et al*., 2000), proof‐of‐concept studies are required to establish the oncogenic roles of endogenous SK4 channels for Ca^2+^ signalling and proliferation and thereby their therapeutic potential in breast cancer.

In order to validate the relevance of SK4 for mammary tumour development *in vivo*, we crossed the well‐established polyoma middle T‐antigen (PyMT) and epidermal growth factor receptor 2 (cNeu) oncogene‐driven mouse breast tumour models to gene‐targeted mice lacking functional SK4 (Guy *et al*., 1992a,b; Sausbier *et al*., 2006). To test whether oncogenic properties of SK4 directly stem from the tumour cells, all animal studies were supported by a parallel analysis of mammary tumour cells derived from PyMT (and cNeu) transgenic tumours in the presence and absence of SK4 channels. While endogenous SK4 channels appear to be molecular classifiers of breast tumour cell growth, SK4 channels present in the tumour stroma may also contribute to the pathogenesis of breast cancer.

## 2 Materials and methods

### 2.1 Mouse models of breast cancer and noninvasive tumour detection and monitoring

Male mammary tumour virus polyoma middle T‐antigen transgenic (MMTV‐PyMT^tg/+^) and male MMTV‐epidermal growth factor receptor 2 (MMTV‐cNeu^tg/+^) mice on a FVB/N background were purchased from The Jackson Laboratory (Guy *et al*., 1992a,b). As genetic background has been identified to be an important modifier of tumorigenesis (Davie *et al*., 2007; Lifsted *et al*., 1998), we backcrossed our previously established SK4 knockout mouse line (SK4 KO; genotype: *SK4* ^*−/−*^) (Shumilina *et al*., 2008) to the FVB/N background for five generations (N5). MMTV‐PyMT^tg/+^ (FVB/N) or MMTV‐cNeu^tg/+^ (FVB/N) mice were then mated with SK4‐deficient animals (N5) in order to generate MMTV‐PyMT^tg/+^ or MMTV‐cNeu^tg/+^ SK4^+/−^ offspring (N6) in high yield as breeders for subsequent crossings. Female MMTV‐PyMT^tg/+^ or MMTV‐cNeu^tg/+^ SK4 WT and respective SK4 KO mice (N6) for the experiments were produced by crossing SK4^+/−^ males carrying either the MMTV‐PyMT^tg/+^ or MMTV‐cNeu^tg/+^ transgene to female mice carrying a heterozygous ablation of SK4 (SK4^+/−^; N6).

Animals were housed in a 12‐h light/dark circle with access to water and food *ad libitum*. All experiments were approved by the local Ethics Committee for Animal Research (Regierungspraesidium Tuebingen). For genotyping, genomic DNA was isolated from tail‐tip biopsies. Primer sets used to detect the MMTV‐PyMT and MMTV‐cNeu transgenes or the different SK4 alleles were as follows: *MMTV‐PyMT forward*: GGA AGC AAG TAC TTC ACA AGG G; *MMTV‐PyMT reverse*: GGA AAG TCA CTA GGA GCA GGG; *MMTV‐cNeu forward*: TTT CCT GCA GCA GCC TAC GC; *MMTV‐cNeu reverse:* CGG AAC CCA CAT CAG GCC: *SK4 forward 1*: TAA GTG CTT GCT GAG TCT GGA; *SK4 forward 2*: CAG GAA GCA CAG GCA CTG C; *SK4 reverse*: AGG AGA GTG ACT GTA GGT GAG.

Starting at an age of 40 or 120 days, tumour‐prone MMTV‐PyMT^tg/+^ or MMTV‐cNeu^tg/+^ SK4 WT and SK4 KO mice, respectively, were palpated weekly until first tumours were identified. Palpation frequency was increased to three times a week after tumour detection and tumour size was measured using a digital calliper. When tumour size had reached a diameter of 15 mm, mice were euthanized with CO_2_ and tissue samples were collected for the subsequent analyses.

### 2.2 Tumour preparation and immunohistochemistry

To guarantee optimal fixation, tumour biopsies were carefully adjusted to a standard size of approx. 7.5 × 7.5 mm (length x width) (Tomayko and Reynolds, 1989) using a scalpel. Fixation was performed for 3 h in 4% paraformaldehyde/PBS (Carl Roth, Karlsruhe, Germany) at room temperature. After several washing steps, standardized tumour samples were cryoprotected using a sucrose gradient starting with 5% sucrose (Carl Roth) for 1 h, followed by 10% sucrose overnight and finally 20% sucrose for 24 h (all diluted in PBS). Upon cryoprotection, tumours were stored at −80°C in Neg‐50™ frozen section medium (Thermo Fisher Scientific, Waltham, MA) for at least 24 h. Prior to the subsequent experiments, tumour tissues were adjusted to −18°C (cutter temperature −21°C), sectioned at 10 μm with a Microm HM 560 (Thermo Fisher Scientific) and mounted on glass slides (Carl Roth). In general, tumour sections derived from SK4 WT and SK4 KO MMTV‐PyMT transgenic tumours, respectively, were mounted together on the same glass slides for further processing. Haematoxylin and eosin staining was performed on sections rehydrated by a series of ethanol according to a previously published protocol (Leiss *et al*., 2011) using Harris haematoxylin solution (Carl Roth) and eosin‐g solution (Carl Roth). After removing excessive dye with ethanol 80% (v/v), tumour sections were dehydrated again with 100% ethanol (v/v) and toluol and finally embedded in DePeX (VWR, Darmstadt, Germany). To identify SK4 channels in tumour biopsies, we adopted an established immunohistochemical method (Bausch *et al*., 2015). In brief, sections were encircled with ImmEdge Hydrophobic Barrier Pen (Vector Laboratories, Burlingame, CA) and then fixed again for 15 min in 4% paraformaldehyde/PBS solution. Unspecific epitopes were blocked for 1 h in blocking solution containing 5% normal goat serum (Vector Laboratories, Burlingame), 1% BSA (Carl Roth), 0.2% glycine (Carl Roth), 0.2% lysine (Carl Roth) and 0.3% Triton X‐100 (Carl Roth) in PBS. Sections were then incubated in primary anti‐SK4 antibody solution (1 : 1000 dilution in blocking solution) overnight at 4°C. The next day, biotinylated anti‐rabbit secondary antibody (1 : 1000 in blocking solution, Vector Laboratories, Burlingame) was applied for 1 h in the dark at room temperature. ABC alkaline phosphatase (AP) solution (Vectastain ABC‐AP‐Kit Standard Alkaline Phosphatase, Vector Laboratories, Burlingame) was used with Vector Blue AP‐substrate (Vector Blue Alkaline Phosphatase Substrate Kit III Vector Laboratories, Burlingame) and levamisole solution (Vector Laboratories, Burlingame) to visualize the antigen–antibody complexes. Stained sections were covered with Aquatex® (Merck Millipore, Darmstadt, Germany), and digital image acquisition was performed using an Axiocam MRcRev.3 camera (Carl Zeiss GmbH, Jena, Germany) attached to an inverse Axiovert 200 M microscope (Carl Zeiss GmbH).

### 2.3 Cell culture of primary MMTV‐cNeu^tg/+^ and MMTV‐PyMT^tg/+^ tumour cells

Tumour biopsies were obtained from SK4‐proficient and SK4‐deficient MMTV‐PyMT^tg/+^ tumours and from MMTV‐cNeu^tg/+^ SK4 WT tumours (diameter of approx. 5 mm) and cut into small pieces with a scalpel and then immediately incubated in a falcon tube containing culture medium [IMEM, 5% fetal bovine serum (FBS), 1% penicillin/streptomycin (Life Technologies, Darmstadt, Germany)] on ice. Under sterile conditions, tumour pieces were then transferred into IMEM (Life Technologies) with 1 mg·mL^−1^ collagenase D (Roche, Mannheim, Germany) at 37°C for 11 min. To achieve optimal dissociation of the tumour tissue, several titration steps supported enzymatic digestion. Cells were then transferred through a 40‐μm cell strainer (BD Biosciences, Heidelberg, Germany) to a petri dish and were grown in monolayer in culture medium [IMEM + 5% FBS and 1% penicillin/streptomycin (Life Technologies)] at 37°C with 5% CO_2_ and 95% relative humidity. Cell concentrations were adjusted, depending on experiment set‐up, from 80 000 to 5 × 10^6^ cells per mL by using 10 μL of the cell suspension for counting in a disposable haemocytometer (Biochrom, Berlin, Germany). We used tumour cells between passages 4 and 13 for all subsequent analyses.

### 2.4 Orthotopic transplantation of murine breast tumour cells

MMTV‐PyMT^tg/+^ SK4 WT or SK4 KO breast cancer cells were orthotopically transplanted into the fourth mammary gland of female FVB/N WT mice (12 weeks of age) (DeNardo *et al*., 2011; Kocaturk and Versteeg, 2015). Briefly, mice were anaesthetized by i.p. injection with 10 μL·g^−1^ body weight of ketamine/xylazine solution (ketamine (10 mg·mL^−1^) and xylazine (1 mg·mL^−1^) in NaCl 0.9%). When sufficient deepness of anaesthesia was confirmed by the loss of pedal reflexes, the experimental mice were fixed on a heating plate (37°C). The area around the fourth mammary gland was shaved and cleaned with ethanol. A short cut of approx. 10 mm was made directly beneath the mammary gland to make it accessible. The gland was cleaned with PBS and fixed with a surgical clamp for the injection of 10^6^ breast cancer cells in PBS (50 μL). Postoperative analgesia was performed with metamizole (200 mg·kg^−1^, s.c.). One week after the procedure, tumour onset was detected by palpation three times a week as described above.

### 2.5 SK4 modulators and chemicals

TRAM‐34 (Wulff *et al*., 2000) was synthesized *in‐house* and dissolved in ethanol (Sigma Aldrich, Taufkirchen, Germany) to a stock solution of 5 mM and then diluted to a final concentration of 0.1, 1, or 10 μM. Apoptosis initiator staurosporine (Cell Signaling/New England Biolabs, Frankfurt, Germany) was dissolved in DMSO to a stock concentration of 1 mM and then diluted to a final concentration of 1 μM in cell culture medium.

### 2.6 Patch clamp recording

On‐cell currents of MMTV‐PyMT^tg/+^ cells were evoked by 41 voltage square pulses (700 ms each) from 0 mV holding potential to voltages between −100 and +100 mV delivered in 5‐mV increments. Cells were superfused at 37°C temperature with NaCl solution (125 mmol·L^−1^ NaCl, 32 mmol·L^−1^ HEPES, 5 mmol·L^−1^ KCl, 5 mmol·L^−1^ D‐glucose, 1 mmol·L^−1^ MgCl_2_, 1 mmol·L^−1^ CaCl_2_, titrated with NaOH to pH 7.4). The pipette solution contained 0 or 0.01 mmol·L^−1^ TRAM‐34 in DMSO, 130 mmol·L^−1^ KCl, 32 mmol·L^−1^ HEPES, 5 mmol·L^−1^ D‐glucose, 1 mmol·L^−1^ MgCl_2_, 1 mmol·L^−1^ CaCl_2_, titrated with KOH to pH 7.4.

We determined the effect of TRAM‐34 in unpaired experiments by comparing macroscopic on‐cell currents recorded with TRAM‐34 in the pipette with those obtained with vehicle alone. A supraeffectively high TRAM‐34 (10 μM) concentration was chosen in order to guarantee a quantitative and instantaneous blockage of SK4 channels. The latter was required because of the high fragility of the giga ohm seal in our experiments. At this high concentration, TRAM‐34 may inhibit other ion channels. The SK4 specificity of the observed TRAM‐34‐sensitive current fraction was therefore estimated by comparing the TRAM‐34 effect between SK4‐proficient and SK4‐deficient cells.

Macroscopic on‐cell currents were analysed by averaging the currents between 100 and 700 ms of each square pulse. Applied voltages refer to the cytoplasmic face of the membrane with respect to the extracellular space. Outward currents, defined as flow of positive charge from the cytoplasmic to the extracellular membrane face, are positive currents and depicted as upward deflections of the original current traces.

### 2.7 mRNA isolation and quantitative real‐time polymerase chain reaction

Isolation of mRNA was performed under RNase‐free conditions based on acid guanidinium thiocyanate/phenol/chloroform extraction as described before (Leiss *et al*., 2011). Tumour samples were homogenized in 1 mL PeqGold RNA pure™ (PeqLab, VWR International, Erlangen, Germany) using an ultraturrax (Dispergieraggregat 1130, Kinematics). Breast tumour cells (5 × 10^6^) at passages 4 to 13 were plated in 55‐cm^2^ petri dishes (Corning, Amsterdam, the Netherlands) and then cultivated at 37°C and 5% CO_2_ for 24 h before G_1_ arrest was induced by serum deprivation (72 h) using IMEM without phenol red (Life Technologies). Arrested cells were either left untreated (t_0_) or were restimulated with culture medium containing 5% FBS in the absence or presence of TRAM‐34 (10 μM) for 0.5, 1.5 or 24 h, respectively. mRNA was isolated from PBS‐washed cells with 1 mL of PeqGold RNA pure™ directly applied to the monolayer. Both tissue‐ and cell‐derived mRNA samples were transferred to 1.5‐mL test tubes and then centrifuged at 13 000 rpm for 10 min at 4°C to remove cell debris. Supernatants were transferred to fresh tubes and then processed according to the manufacturer's recommendations. mRNA concentrations were determined using a nanophotometer (Implen, München, Germany). DNA contaminations were removed by a DNase digestion step by adding 5 μL of DNase (Roche) and 6 μL of DEPC‐treated water per 50 μL sample. Digestion was allowed for 30 min at 37°C and followed by enzyme inactivation for 5 min at 80°C. For subsequent analyses, mRNA samples were adjusted to a final concentration of 0.1 μg·μL^−1^ in DEPC‐treated water. Tumour tissue‐ or tumour cell‐derived mRNA (5 μL) was mixed with iScript™ (4 μL; Bio‐Rad, München, Germany) and DEPC‐treated water (10 μL) in the absence or presence of reverse transcriptase (1 μL) for cDNA synthesis for 30 min at 42°C in a MasterCycler (Eppendorf, Hamburg, Germany). After heat inactivation of the reverse transcriptase (RT), cDNA was diluted with 180 μL DEPC‐treated water to a final volume of 200 μL. Quantitative real‐time polymerase chain reaction (qPCR) was performed using 2 μL of the diluted cDNA with SYBR^®^ green Kit (Bio‐Rad, München, Germany) and specific primer pairs. SYBR^®^ green fluorescence was detected with Opticon™ (MJ Research, St. Bruno). Cycle of time values (C_t_) were determined with Opticon Monitor™ analysis software version 1.4 from triplicate. C_t_ values of genes of interest were then subtracted from C_t_ values of the simultaneously amplified housekeeping gene (β‐actin) to obtain ΔC_t_ values. 2 raised by the exponent of negative ΔC_t_ value represents the relative mRNA level to the corresponding housekeeper β‐actin. Primers were designed with Primer3web version 4.0.0, Ensembl and purchased from *Eurofins MWG GmbH, Ebersberg*. Primers used to determine the expression of SK1‐4, cyclin D1 (CycD1), c‐fos, c‐jun and β‐actin were as follows: *SK1 forward*: TGT ACC ACG CCC GAG AGA TC; *SK1 reverse*: TCC AGC GAG ATC AGG GAC AC; *SK2 forward*: TCT CTC CAC GAT CAT CCT GCT; *SK2 reverse*: CTG CTC CAT TGT CCA CCA TGA; *SK3 forward*: CGC CTA TCA CAC AAG GGA AGT; *SK3 reverse*: ACG CTC GTA GGT CAT GGC TA; *SK4 forward*: ATG TGG GGC AAG ATT GTC TG; *SK4 reverse*: GTG TTT CTC CGC CTT GTT GA; *CycD1 forward*: CCT TTG TGG CCC TCT GTG C; *CycD1 reverse*: CTC CCA GCA GCT ACC ATG GA; *c‐fos forward*: ACT ACC ATT CCC CAG CCG AC; *c‐fos reverse*: TCT GCG CAA AAG TCC TGT GT; *c‐jun forward*: CCA AGA ACG TGA CCG ACG AG; *c‐jun reverse*: GCG TGT TCT GGC TAT GCA GT; *β‐actin forward*: GAC GGC CAG GTC ATC ACT AT; *β‐actin reverse*: CCA CAG GAT TCC ATA CCC AAG.

### 2.8 Impedance and grid‐based proliferation assays

Proliferation was assessed with μ‐dishes grid‐500 (ibidi^®^, Planegg/Martinsried, Germany) and xCelligence system allowing for continuous measurement by impedance readout (Steinle *et al*., 2011). For the grid assay, cells were passaged and counted as described. Cell suspension was adjusted to 80 000 cells per grid and cells were allowed to attach to the bottom of the petri dish for 24 h at 37°C, 5% CO_2_ before serum was withdrawn to induce G_1_ arrest. Restimulation with culture medium (IMEM + 5% FBS and 1% Pen/Strep, Life Technologies) was performed after 72 h using either vehicle or compounds as indicated. Every 24 h, digital pictures of previously defined areas were taken for four consecutive days. Cell number at every time point was determined using ImageJ software (imagej 1.46).

### 2.9 Ki‐67 proliferation analysis

Cells were seeded in 12‐well chambers (Ibidi, Martinsried, Germany) in duplicate at densities of 80 000, 50 000 or 30 000 cells per well and grown for 24, 48 or 72 h, respectively. For assessing proliferation with immunofluorescence, cells were fixed with 70% ethanol at −20°C for 10 min, washed three times with PBS and incubated in PBS with 0.1% Triton X‐100 for 15 min. Then, cells were washed three times with PBS and blocked with 10% normal donkey serum in PBS (Dianova, Hamburg, Germany) for 1 h. Cells were then incubated in a 1 : 1000 diluted anti‐Ki‐67 rabbit primary antibody solution (Cell Signaling Technologies/New England Biolabs, Frankfurt, Germany) in 1.5% normal donkey serum in PBS for 2 h. After washing three times with PBS, cells were incubated in a 1 : 800 diluted Alexa Fluor^®^ 555 donkey anti‐rabbit secondary antibody solution (Invitrogen, Carlsbad, CA) in 1.5% normal donkey serum in PBS for 1 h. Cells were washed another three times with PBS and coverslipped with DAPI‐containing Vectashield antifade mounting medium (Vector laboratories, Lorrach, Germany). For each chamber, four microscopy sections were taken and Ki‐67‐positive cell count was related to the total number of cells in the section.

### 2.10 Cell cycle analysis by flow cytometry

For flow cytometry, a well‐established protocol to stain DNA with propidium iodide (PI) was used (Pozarowski and Darzynkiewicz, 2004). 1.5 × 10^6^ cells were seeded into a petri dish of 55‐cm^2^ area (triplicate for every condition) and cultivated for 24 h at 37°C, 5% CO_2_. To induce G_1_ arrest, cells were washed twice with PBS and serum was withdrawn (Life Technologies) for 72 h at 37°C, 5% CO_2_. Restimulation was performed with culture medium for 24 h. After trypsinization and counting, cell number was adjusted with PBS to 1 × 10^6^ cells per 0.5 mL. 0.5 mL of the cell suspension was mixed with 4.5 mL of 70% ethanol (v/v) for fixation and samples were stored at 4°C. During cell counting, we did not find evidence for cells stacked or grouped together. PI staining solution (10 μg·mL^−1^) with RNase (100 μg·mL^−1^) to avoid false RNA staining was added to the singularized cells at room temperature for 30 min. Samples that were incubated in staining solution without PI were included as negative controls. A FACSCanto flow cytometer (BD Biosciences) and the FACSDiva software package (BD Biosciences) were used for data acquisition. Disproportions between height, width and area as indicators of doublets, cell clumps and/or debris were excluded from these analyses by using respective scatter gates. Evaluation of DNA content was carried out with FlowJo 7.6.1 (*TreeStar*) using Watson curve‐fitting algorithm to estimate the number of cells in G_1_, S and G_2_/M phases of the cell cycle.

### 2.11 Calcium imaging using FURA‐2‐AM

Intracellular calcium [Ca^2+^]_i_ of murine breast tumour cells was determined with chelating agent FURA‐2‐AM, which can be excited at 340 and 380 nm and the ratio of the emissions at these wavelengths (FL 340/380) corresponds to [Ca^2+^]_i_. For [Ca^2+^]_i_ measurements, 80 000 cells were plated onto a CELLview™ culture dish (Greiner Bio‐One, Frickenhausen, Germany). For acute calcium imaging, cells were cultivated for 24 h at 37°C and 5% CO_2_ followed by serum withdrawal. After 72 h, cells were washed with 2 mL Ca^2+^‐free incubation buffer and then loaded with 1 μM FURA‐2‐AM (Calbiochem/Merck KGaA, Darmstadt, Germany) diluted in 1 mL incubation buffer for 45 min. Upon uptake, FURA‐2‐AM is cleaved by intracellular esterases into free FURA‐2, which can bind [Ca^2+^]_i_. For imaging of spontaneous calcium signals, cells were cultivated for 24, 48 or 72 h after plating, respectively. Then, cells were washed with 2 mL of incubation buffer containing 1.8 mM Ca^2+^ and loaded with 1 μM FURA‐2‐AM in 1 mL incubation buffer (1.8 mM Ca^2+^). [Ca^2+^]_i_ was measured with VisiView (Visitron, Puchheim, Germany) and Spot Inside camera (Visitron) attached to an inverse microscope (Axiovert S100, halogene lamp XBO 75, Carl Zeiss, Jena, Germany) for a total of 20 min under different conditions as indicated in the corresponding experiments. Every two‐seconds, digital pictures were taken at 340 nm for FURA‐2 in the Ca^2+^‐bound form and 380 nm for free FURA‐2. Fluorescence intensity of the single cells of every picture sequence was determined with ImageJ software 1.46.

### 2.12 Isolation of cytosolic and mitochondrial fractions from breast tumour cells

Cells (5 × 10^6^) were maintained at 37°C and 5% CO_2_ for 24 h before treatment with vehicle, staurosporine (1 μM) (STS, Cell Signaling Technologies/New England Biolabs) or SK4 inhibitor TRAM‐34 (10 μM), respectively. Ten cell culture dishes of 55‐cm^2^ area were necessary per condition. After several time points, cells were washed, trypsinized and washed in ice‐cold PBS with 1 μM Na_3_VO_4_ (in demineralized water, Sigma Aldrich) twice. After a centrifugation step at 1000 rpm, the cell pellet was resuspended in a fivefold amount of isolation buffer containing 1 tablet of cOmplete Mini, EDTA free (Roche). For homogenization, a combination of manual (30 s) and electric homogenization steps with an ultraturrax (30 s) was used as this guaranteed best separation of mitochondrial and cytosolic fraction. To isolate the purified mitochondria, a series of centrifugation steps was performed: Initial centrifugation for 10 min at 1000 ***g*** removed the nuclei and cell residues, whereas mitochondrial and cytosolic fractions in the supernatant were transferred to a fresh microtube and centrifuged again for 15 min at 14 000 ***g*** resulting in the mitochondrial pellet. Supernatant containing cytosolic fraction was carefully transferred into a fresh microtube for protein quantification, whereas the mitochondrial fraction was washed twice with 250 μL of isolation buffer and finally dissolved in 25 μL of isolation buffer. Protein determination was performed using Lowry's method with Total Protein Kit, Micro‐Lowry, Peterson's Modification (Sigma Aldrich). Samples were used immediately or stored at −80°C until SDS/PAGE and western blot analyses were performed.

### 2.13 SDS/PAGE and western blot analyses

SDS/PAGE gels containing 17.5% acrylamide (Carl Roth) were used for separating 40 μg of the mitochondrial or cytosolic protein fractions. Before loading, protein samples were denatured in 4 × Laemmli protein buffer for 10 min at 95°C. Protein ladder IV (PeqLab, VWR, Darmstadt, Germany) was used as protein standard allowing for an accurate determination of protein sizes. Electrophoresis was performed at 80–120 V for 90 min. For western blot, proteins of both fractions were transferred onto Membrane Immobilon^®^ Millipore PVDF (Carl Roth) by using a semidry blotting system (Carl Roth) at 80 mA for 1 h followed by 150 mA for 15 min per membrane.

To block unspecific bindings, membranes were incubated in 5% milk powder (Carl Roth) in 1x TBST, which was diluted with demineralized water from 10x TBST (Tris 12.1 g (Carl Roth), NaCl 82.3 g (Carl Roth), Tween‐20 5 mL (Serva Electrophoresis, Heidelberg, Germany) and demineralized water 1000 mL). After three washing steps for 10 min with 1x TBST, membranes were incubated in the primary antibody solution containing 5% BSA (Carl Roth), 0.05% NaN_3_ (Carl Roth) in 1x TBST on a rotor at 4°C overnight. The next day, primary antibody–antigen complexes were detected by appropriate secondary antibody conjugated to fluorescent dyes (in 1x TBST) for 1 h at room temperature. Signals were detected with an Ettan Dige Imager (GE Healthcare, München, Germany), and protein amount was analysed with Image Quant TL 7.0 software (GE Healthcare). Antibodies and dilutions used were as follows: anti‐cytochrome c (1 : 200) (Cell Signaling Technologies/New England Biolabs); anti‐α‐tubulin (1 : 1000) (Cell Signaling Technologies/New England Biolabs); anti‐HSP 60 (1 : 200) (*Santa Cruz*); anti‐GAPDH (1 : 1000) (Cell Signaling Technologies/New England Biolabs); anti‐COX IV (1 : 1000) (Cell Signaling Technologies/New England Biolabs), anti‐caspase 3 (1 : 1000) (Cell Signaling Technologies/New England Biolabs); anti‐cleaved caspases 3 (1 : 1000) (Cell Signaling Technologies/New England Biolabs); anti‐rabbit ECL Plex Cy5 (1 : 2000) (GE Healthcare); anti‐mouse ECL Plex Cy3 (1 : 2000) (GE Healthcare).

### 2.14 Statistics

Statistical analyses were performed with Microsoft Excel and GraphPad Prism. Graphs show mean ± SE for every condition. Two groups were compared by an unpaired *t*‐test; if three or more groups were compared, a one‐way ANOVA with following Bonferroni correction was performed. For Kaplan–Meier analyses, a log‐rank test was used and *P*‐values were calculated from χ^2^ (one degree of freedom).

## 3 Results

### 3.1 Murine SK4 channels in breast tumours and tumour cells derived thereof

**Figure 1:**
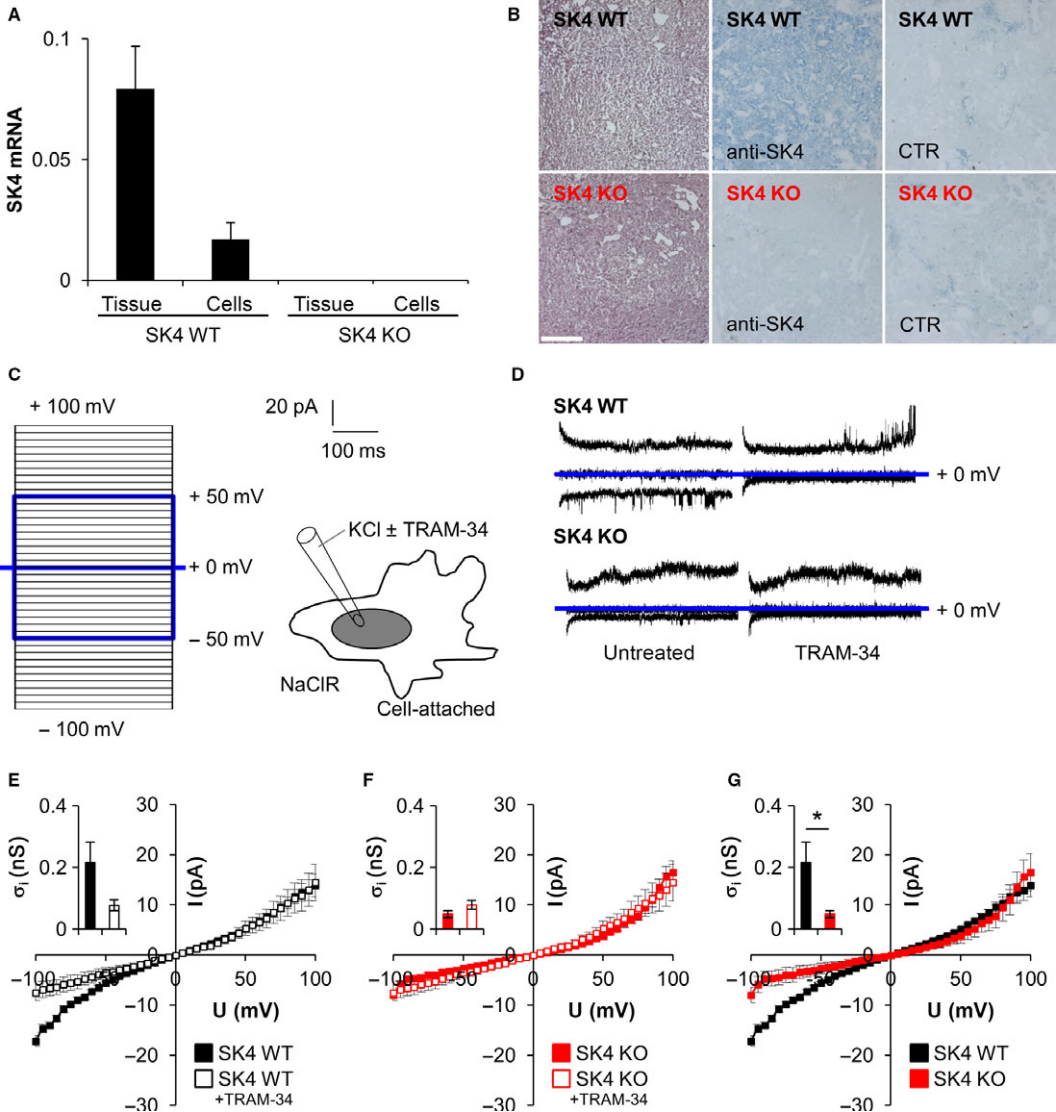
Expression and functional analysis of SK4 channels in MMTV‐PyMT^tg/+^ breast tumours and tumour‐derived cells. (A) MMTV‐PyMT^tg/+^ tumour tissue (*n* = 7) and cells (*n* = 4) express high levels of SK4 mRNA. β‐Actin was coamplified in each sample and used as a reference for normalization of mRNA abundance. As expected, no SK4 mRNA was detectable in SK4 KO tissue and cells (*n* = 2). (B) Haematoxylin and eosin staining of MMTV‐PyMT^tg/+^ SK4 WT and SK4 KO tumours (upper and lower left panels panel). Immunohistochemical detection of SK4 channels in PyMT transgenic SK4 WT tumours using a SK4‐specific primary antibody (upper middle panel). As expected, SK4‐negative tumours remained unstained (lower middle panel). The specificity of the immunohistochemical approach was further controlled by staining of SK WT and SK4 KO tumours in the absence of the primary antibody (upper and lower right panels) (*n* = 3 for each genotype; scale bar = 500 μm). (C) Macroscopic on‐cell currents were recorded from MMTV‐PyMT^tg/+^ tumour‐derived SK4 WT and SK4 KO cells using a KCl pipette and NaCl bath solution applying the depicted pulse protocol. (D) On‐cell current tracings recorded during voltage square pulses (C) to ‐50, 0 and +50 mV. Currents were recorded from MMTV‐PyMT^tg/+^ SK4 WT (top) and SK4 KO (bottom) cells with (right) and without (left) TRAM‐34 (10 μM) in the pipette solutions. (E–G) Dependence of the mean macroscopic on‐cell currents on clamp voltage in MMTV‐PyMT^tg/+^ SK4 WT (E, G) and SK4 KO (F, G) cells recorded in the absence (closed squares) and presence (open squares) of TRAM‐34 in the pipette. Inlays show inward conductance as calculated from the data in (E) and (F) by linear regression of MMTV‐PyMT^tg/+^ SK4 WT and SK4 KO cells in the absence (closed bars) and presence (open bars) of TRAM‐34 (*P < 0.05, unpaired two‐tailed Welch‐corrected Student's *t*‐test) (*n* = 11/7; 17/15; 11/15). SK4, calcium‐activated potassium channel with intermediate conductance; MMTV, mouse mammary tumour virus; PyMT, polyoma virus middle T‐antigen; WT, wild‐type; KO, knockout; CTR, control; mV, millivolt; pA, picoampere; KCl, potassium chloride; TRAM‐34, triarylmethan‐34; NaCl, sodium chloride; I, current; U, voltage.

We investigated the PyMT‐ and cNeu‐driven *in vivo* mouse models of breast tumour development for their SK4 expression status (Fig. 1A and 1Fig. S1A). We found significantly higher levels of SK4 mRNA (34.5‐fold) in tumour biopsies of MMTV‐PyMT^tg/+^ mice compared to biopsies derived from nonmalignant mammary glands of FVB/N wild‐type animals. (Fig. S1B), suggesting that spontaneous formation and progression of breast tumours via oncogenic PyMT pathways may require functional SK4 channels. We also compared SK4 mRNA expression to that of phylogenetically related SK channels in breast tumour samples revealing the highest levels for SK4 in this model (Fig. 1C,D). In order to establish the endogenous role of SK4 in the MMTV‐driven transgenic mouse models, we crossed the MMTV‐PyMT^tg/+^ and MMTV‐cNeu^tg/+^ animals, respectively, to a *KCNN4* gene‐targeted SK4 knockout mouse line (SK4 KO) on a FVB/N background. SK4‐deficient MMTV‐PyMT^tg/+^ and MMTV‐cNeu^tg/+^ animals were born at the expected Mendelian ratios.

Analysis of primary cultures established from dissociation of multiple MMTV‐PyMT^tg/+^ breast tumours confirmed SK4 expression in tumour‐derived cells, although at relatively lower levels compared to the respective tumours *in situ* (Fig. 1A). As expected, corresponding samples from MMTV‐PyMT^tg/+^ SK4 KO animals were negative for SK4 mRNA (Fig. 1A). Immunostaining of MMTV‐PyMT^tg/+^ breast tumour tissue using SK4‐specific antibodies further confirmed SK4 protein abundance in transformed mouse mammary epithelium (Fig. 1B). Antibody specificity for the SK4 epitopes was verified by parallel analysis of breast tumours derived from MMTV‐PyMT^tg/+^ SK4 KO animals (Fig. 1B). To determine the functionality of SK4 channels in primary breast tumour cells, plasma membrane K^+^ currents were recorded with the cell‐attached patch clamp technique (Fig. 1C,D). Currents were elicited in cells in the presence and absence of the SK4 inhibitor TRAM‐34 (10 μM) by voltage square pulses ranging from −100 to +100 mV (Fig. 1C). TRAM‐34‐sensitive inward currents were observed in MMTV‐PyMT^tg/+^ SK4 WT‐derived breast tumour cells (Fig. 1D, upper tracings and Fig. 1E). Importantly, TRAM‐34, albeit at very high concentration, did not affect inward currents of the SK4‐deficient primary breast tumour cells (Fig. 1D, lower tracings and Fig. 1F). In accordance with an SK4‐generated current fraction in MMTV‐PyMT^tg/+^ tumour cells, inward currents and calculated inward conductance were significantly lower in cells lacking endogenous SK4 channels (Fig. 1G, red symbols/column) than in SK4‐proficient cells (Fig. 1G, black symbols/column). The SK4‐dependent current fraction (difference between red and black symbols in Fig. 1G) exhibited strong inward rectification as reported for canonical SK4 channels which are usually present at the plasma membrane of, for example, endothelial cells and smooth muscle cells (Grgic *et al*., 2005; Toyama *et al*., 2008).

### 3.2 Function of SK4 channels in growth and cell cycle control

**Figure 2:**
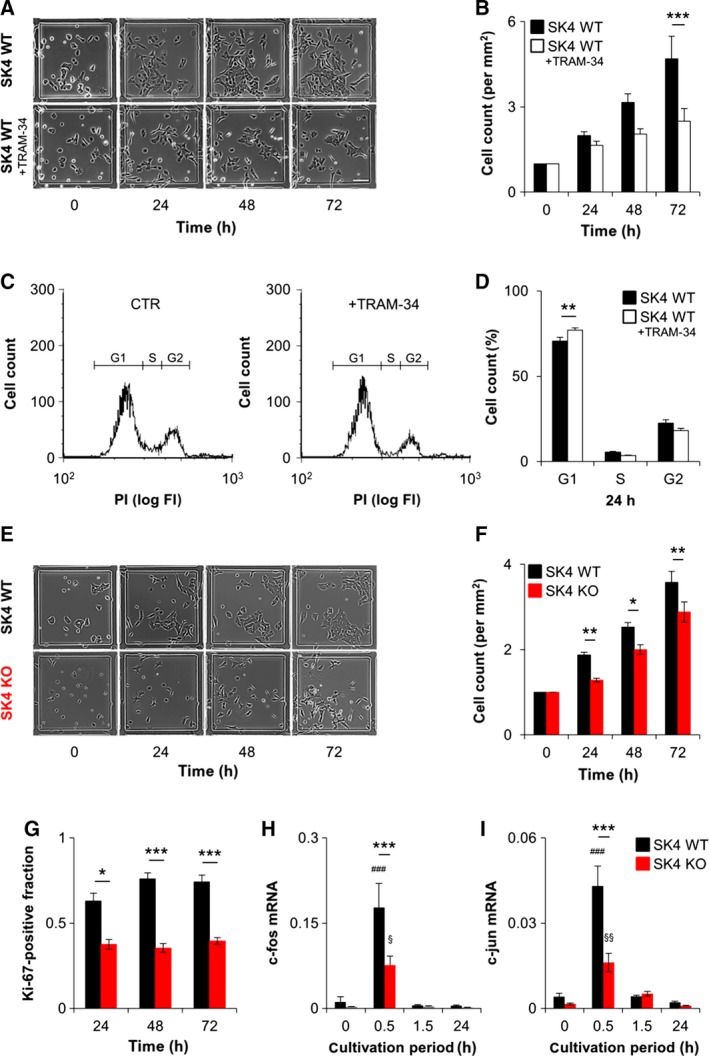
Proliferation of MMTV‐PyMT^tg/+^breast tumour cells requires functional SK4 channels. (A) Effect of TRAM‐34 (10 μM) or vehicle (CTR) on the growth of MMTV‐PyMT^tg/+^ SK4 WT cells. Representative pictures were acquired at the different time points indicated in the mini‐grid assay (scale bar = 100 μm). (B) Cells depicted in (A) were counted with imagej software version 1.46 and cell numbers were normalized to t_0_ for each time point and treatment (*n* = 10). One‐way ANOVA followed by Bonferroni correction was used to test for statistical significance (***P < 0.001). (C) Representative flow cytometry of MMTV‐PyMT^tg/+^ SK4 WT cells treated for 24 h with vehicle (CTR) or TRAM‐34 (10 μM). DNA content was visualized with propidium iodide. (D) Flow cytometry analysis reveals a significantly higher amount of TRAM‐34‐treated MMTV‐PyMT^tg/+^ SK4 WT cells (open bars) in G_1_ and a decreased number of cells in S phase as compared to vehicle‐treated cells (black bars) indicating cell cycle arrest after SK4 blockade (*n* = 4). One‐way ANOVA followed by Bonferroni correction was used to test for statistical significance (*P < 0.05, **P < 0.01). (E) Representative mini‐grid pictures of MMTV‐PyMT^tg/+^ SK4 WT and SK4 KO cells at 0 and 72 h after serum restimulation (scale bar = 100 μm). (F) Mean cell number (*n* = 31) counted from the data in (E) with imagej software version 1.46. Cell numbers were normalized to t_0_ for each time point and genotype. Statistical analysis was performed by one‐way ANOVA followed by Bonferroni correction (**P < 0.01). (G) Ki‐67 immunofluorescence indicated the fraction of proliferative WT and SK4 KO cells after growth for 24, 48 or 72 h (*n* = 6). Statistical analysis was performed by one‐way ANOVA followed by Bonferroni correction (*P < 0.05, ***P < 0.001). (H–I) Analysis of G_1_‐phase cell cycle markers c‐fos and c‐jun in MMTV‐PyMT^tg/+^ SK4 WT (blacks bars) and SK4 KO (red bars) cells. Cells were arrested in G_1_ phase by serum withdrawal and were then restimulated with serum‐containing medium for the indicated cultivation periods (*n* = 4–5). Statistical analysis by one‐way ANOVA followed by Bonferroni correction (***P < 0.001). SK4, calcium‐activated potassium channel with intermediate conductance; WT, wild‐type; TRAM‐34, triarylmethan‐34; CTR, control; KO, knockout; PI, propidium iodide; FI, fluorescence intensity.

We next examined whether SK4 activity plays a role in the growth factor‐induced proliferation of PyMT breast cancer cells. Cell growth was assessed quantitatively by a mini‐grid‐based set‐up (Fig. 2A,B). Cell numbers were determined after 72 h of serum starvation (t_0_), and following readdition of serum every 24 h thereafter for four consecutive days by taking micrographs of previously defined areas of the cell layer. Cell counts upon pharmacological inhibition of SK4 using 10 μM TRAM‐34 showed a tendency to lower numbers at all time points analysed (Fig. 2B). Importantly, this difference reached statistical significance at 72 h for PyMT transgenic cells derived from five different SK4 WT tumours treated with 0.1 to 10 μM TRAM‐34 (Fig. 2B and Fig. S2A). Accordingly, cell cycle analyses using propidium iodide to quantify the amount of DNA suggest a G_1_/S arrest in PyMT transgenic breast tumour cells after SK4 inhibition (Fig. 2C,D), whereas apoptotic parameters such as cytochrome c release and caspase 3 activation were not affected by TRAM‐34 (Fig. S1E). In line with the pharmacological blockade of SK4 channels in MMTV‐PyMT^tg/+^ SK4 WT cells, lack of endogenous SK4 channels attenuated the growth of serum‐restimulated breast tumour cells as well as the antigrowth effects of TRAM‐34 (Fig. 2E,F and Fig. S2B). This essential role of SK4 as regulator of growth was further substantiated by a significantly lower number of Ki‐67‐positive nuclei in SK4 KO cells compared to WT cells (Fig. 2G and Fig. S3). Finally, a reduced response of the G_1_ cell cycle phase markers *c‐fos* and *c‐jun* (Fig. 2H,I) to acute serum restimulation in PyMT transgenic SK4 KO cells confirmed the role of SK4 for the control of cell growth, that is replication and division.

### 3.3 Modulation of intracellular Ca^2+^ levels by SK4 activity

**Figure 3:**
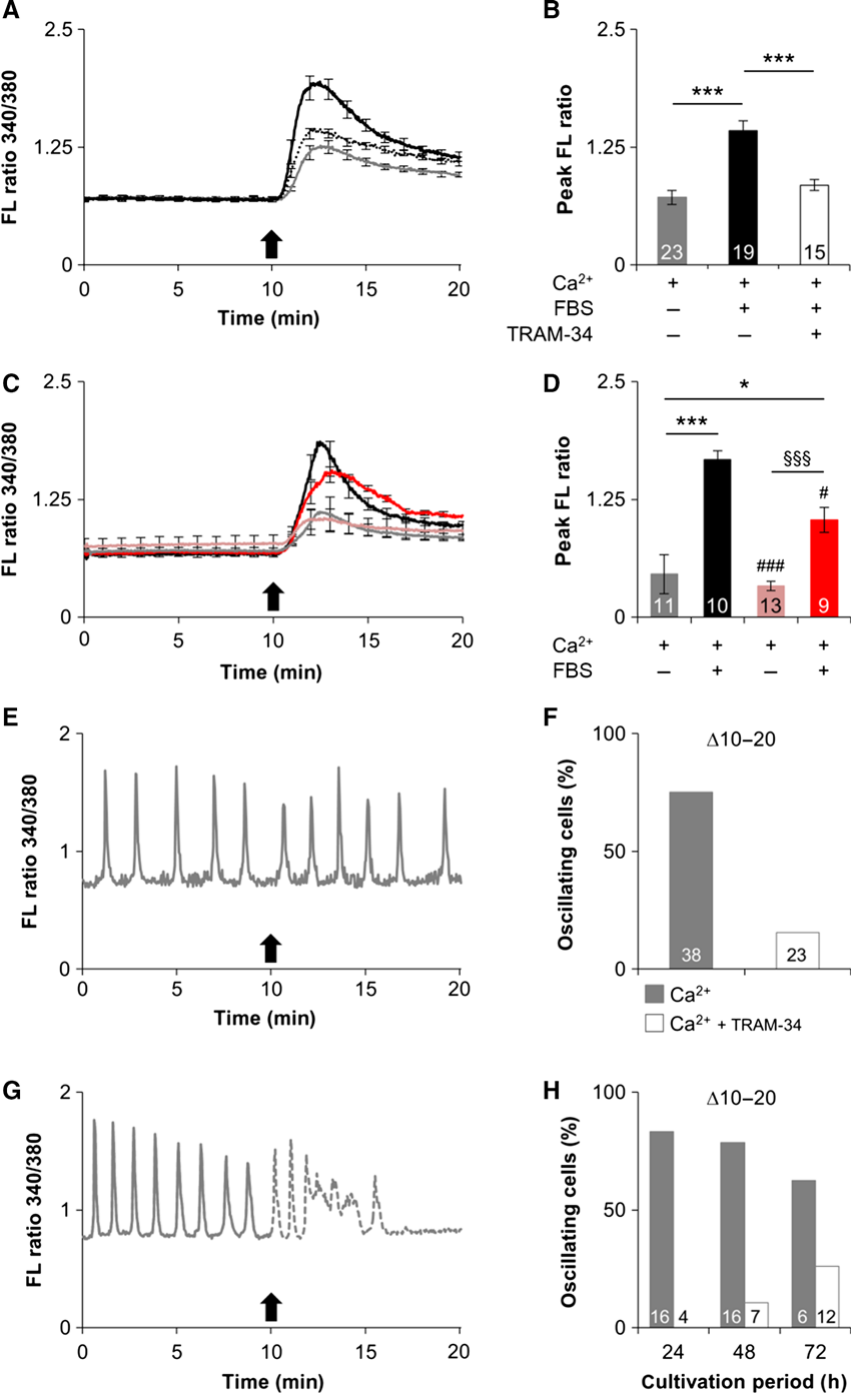
SK4 channels control [Ca^2+^]_i_ signals in MMTV‐PyMT^tg/+^ breast tumour cells. (A‐B) Acute Ca^2+^ signals of MMTV‐PyMT^tg/+^ mammary tumour cells in the presence or absence of the SK4 inhibitor TRAM‐34. FURA‐2‐AM‐loaded cells were monitored in Ca^2+^‐free buffer for 10 min and then treated with incubation buffer containing Ca^2+^ (1.8 mM) only or Ca^2+^ (1.8 mM) plus FBS (5%) plus TRAM‐34 (0 or 10 μM) as indicated. Arrow indicates buffer replacement. (B) The mean maximal FL ratio of the 340/380‐nm wavelength was determined relatively to the respective baseline FL ratio [statistical analysis by one‐way ANOVA followed by Bonferroni correction (***P < 0.001)]. (C–D) Acute Ca^2+^ signals of MMTV‐PyMT^tg/+^ SK4 WT and SK4 KO mammary tumour cells. FURA‐2‐AM‐loaded cells were monitored in Ca^2+^‐free buffer for 10 min and then treated with incubation buffer containing Ca^2+^ (1.8 mM) only or Ca^2+^ (1.8 mM) plus FBS (5%) ±TRAM‐34 as indicated. Arrow indicates buffer change. (D) The mean maximal FL ratio of the 340/380‐nm wavelength was determined relatively to the respective baseline FL ratio. Statistical analysis by one‐way ANOVA followed by Bonferroni correction (**P < 0.01, ***P < 0.001 and ^#^ *P* < 0.05 or ^###/§§§^ *P* < 0.001 for the comparison to the SK4 WT group stimulated with Ca^2+^ and FBS ). (E–G) Spontaneous Ca^2+^ signals in MMTV‐PyMT^tg/+^ breast tumour cells in the absence or presence of the specific SK4 inhibitor TRAM‐34. Cells were cultivated for 24, 48 or 72 h and then loaded with FURA‐2‐AM in incubation buffer containing Ca^2+^ (1.8 mM). After monitoring Ca^2+^ oscillations for 10 min, cells were superfused with Ca^2+^‐containing incubation buffer in the (E) absence or (G) presence of TRAM‐34. Representative traces are shown for *t* = 48 h. Arrows indicate buffer change. (F) Percentage of all cells with Ca^2+^ oscillations during the first 10 min that continued to show alternations in [Ca^2+^]_i_ in the next 10 min after adding TRAM‐34 or saline. (H) Percentage of initially oscillating cells at 24, 48 or 72 h after plating that continued to oscillate after the buffer change from 10 to 20 min ±TRAM‐34. FL, fluorescence; FBS, fetal bovine serum; TRAM‐34, triarylmethan‐34; MMTV, mouse mammary tumour virus; PyMT, polyoma virus middle T‐antigen; SK4, calcium‐activated potassium channel with intermediate conductance; FURA‐2‐AM, FURA‐2‐acetoxymethyl ester.

Intracellular Ca^2*+*^ signals are associated with different phases of cell cycle progression (Roderick and Cook, 2008; Whitfield *et al*., 1995). Elevated [Ca^2+^]_i_ and consecutive SK4 activation result in hyperpolarization, which, in turn, may favour the entry of Ca^2+^ by increasing its electrochemical driving force across the plasma membrane or by keeping voltage‐dependent Ca^2+^ entry pathway in an active and/or highly conductive state. To assess whether SK4 controls the Ca^2+^ dynamics of PyMT transgenic breast tumour cells, growth factor‐induced Ca^2+^ responses were determined in the presence and absence of TRAM‐34. Changes in [Ca^2+^]_i_ were monitored after serum starvation in FURA‐2‐AM‐loaded cells by changing the Ca^2+^‐free loading buffer to a buffer containing Ca^2+^ (1.8 mM) in the presence and absence of FBS. Ca^2+^ superfusion alone resulted in a transient [Ca^2+^]_i_ rise showing an incomplete decay over 10 min (Fig. 3A). FBS augmented the Ca^2+^ buffer‐induced [Ca^2+^]_i_ amplitude of PyMT transgenic breast tumour cells (Fig. 3A,B), which is in line with findings from Lepple‐Wienhues *et al*. (1996). Conversely, TRAM‐34 suppressed the FBS‐induced fraction of the [Ca^2+^]_i_ response to levels observed under FBS‐free conditions, suggesting that changes in SK4 activity are relevant for growth factor‐dependent control of [Ca^2+^]_i_ homeostasis (Fig. 3A,B). Accordingly, we observed a significantly lower FBS‐induced [Ca^2+^]_i_ amplitude in SK4‐deficient PyMT transgenic breast tumour cells (Fig. 3C,D). In the next series of experiments, spontaneous changes of [Ca^2+^]_i_ were investigated at different time points in nonarrested PyMT breast cancer cells. In general, about 4–20% of the cells (*n* = 726) displayed spontaneous [Ca^2+^]_i_ transients with different amplitude and frequency (Fig. 3E–H). Spontaneous collapse of these [Ca^2+^]_i_ transients during Ca^2+^ imaging was observed in 17%, 21% and 38% of the cells at 24‐, 48‐ and 72‐h cultivation time, respectively. SK4 inhibition by TRAM‐34, however, raised these values to 100%, 90% and 74% at the time points analysed (Fig. 3F,H), again indicating the functional significance of SK4 for Ca^2+^ signalling in PyMT transgenic breast tumour cells.

### 3.4 Impact of SK4 channels on survival time in breast cancer mouse models

**Figure 4:**
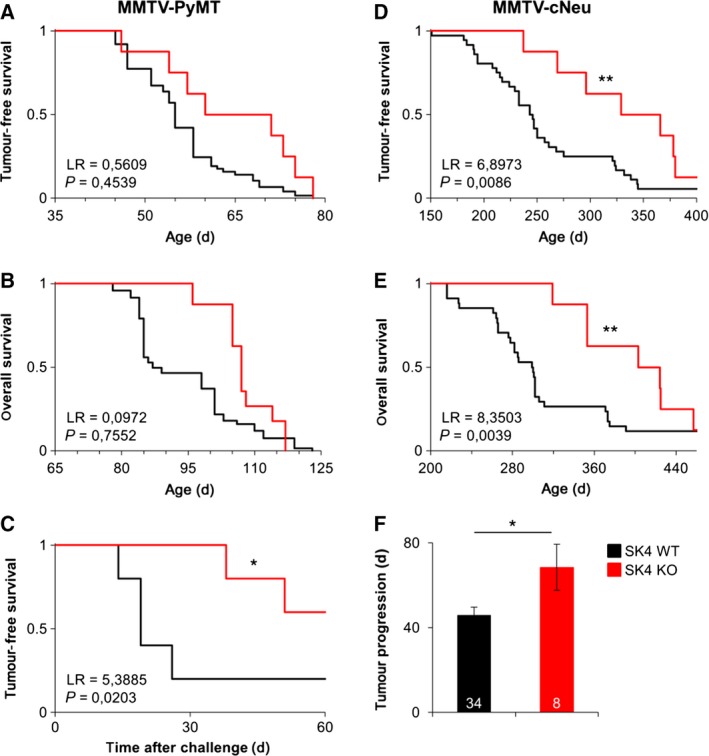
Kaplan–Meier analysis of MMTV‐PyMT^tg/+^ and MMTV‐cNeu^tg/+^ SK4 WT and SK4 KO mice. (A–B) Tumour‐free survival (*n* = 25/8) and overall survival (*n* = 19/8) of MMTV‐PyMT^tg/+^ mice (FVB/N background) with a genetic ablation of SK4 compared to SK4 WT littermates. (C) Tumour‐free survival of FVB/N WT mice that were orthotopically transplanted with MMTV‐PyMT^tg/+^ SK4 WT (mean survival 20 ± 2 days) or SK4 KO (mean survival 45 ± 7 days) breast tumour cells, respectively (*n* = 5). (D–E) Tumour‐free and overall survival of MMTV‐cNeu^tg/+^ mice (FVB/N background) with a genetic ablation of SK4 (322 ± 21 days and 391 ± 19 days, respectively) compared to SK4 WT littermates (247 ± 9 days and 291 ± 9 days, respectively) (*n* = 8/34). (Statistical analysis for A‐E was performed by the log‐rank test followed by *P*‐value calculation from χ^2^ with one degree of freedom (**P < 0.01, ***P < 0.001). (F) Tumour progression of MMTV‐cNeu^tg/+^ SK4 WT (46 ± 4 days) and SK4 KO (68 ± 11 days) mice. Tumour progression was defined as the time between first palpation of a tumour until abort criterion (15‐mm‐diameter tumour size) was reached. Statistical analysis was performed by an unpaired two‐tailed Student's *t*‐test (*P < 0.05). SK4, calcium‐activated potassium channel with intermediate conductance; WT, wild‐type; KO, knockout; MMTV, mouse mammary tumour virus; PyMT, polyoma virus middle T‐antigen; LR, log‐rank value.

Our *in vitro* data on the role of SK4 in cell growth, cell cycle control and Ca^2+^ signalling collectively point to an antitumour effect of SK4 inhibition *in vivo*. Performing Kaplan–Meier analyses, we demonstrate that neither tumour‐free survival nor overall survival of breast cancer‐developing MMTV‐PyMT^tg/+^ mice was significantly affected by the global lack of SK4 (Fig. 4A,B). Median survival rates of the SK4 KO mice tend to result in a better outcome, but this tendency did not reach statistical significance. As SK4 channels are known to be important determinants in the activation of T and B cells (Begenisich *et al*., 2004; Kang *et al*., 2014; Xu *et al*., 2014), decreased tumour formation in SK4 KO mice may be masked by the inability of the SK4‐deficient immune system to recognize and/or control tumorigenesis in breast cancer. To prove this hypothesis, we took a different approach and established an orthotopical transplantation model. *In vitro* propagated breast cancer cells derived from tumour‐bearing PyMT transgenic SK4 WT or SK4 KO mice were allowed to grow into the same origin site as the primary tumours by their implantation into the mammary fat pad of SK4‐proficient FVB/N wild‐type mice. Beginning 14 days postinjection, FVB/N wild‐type hosts that had received SK4 WT cells developed palpable tumours, whereas animals implanted with SK4 KO cells exhibited a significant longer tumour‐free survival time (Fig. 4C).

The MMTV‐PyMT model usually develops multiple tumours in asynchronous manners that are spreading to different organs. As the impact of stromal and/or immune cell SK4 channels on tumour development might differ between highly malignantly and slowly progressing tumours, we analysed whether SK4 activity affects the survival rates in an alternative mouse model of spontaneously occurring tumours, that is the MMTV‐cNeu^tg/+^ breast cancer model (Guy *et al*., 1992b). This model develops monofocal breast tumours with a later onset and a slower tumour progression than the MMTV‐PyMT model. Notably, tumour‐free survival and overall survival were significantly prolonged in MMTV‐cNeu^tg/+^ mice lacking SK4 channels (Fig. 4D,E) as compared to the SK4 WT mice. Moreover, the time span between first tumour palpation and reaching of the abort criterion as a measure of tumour growth was significantly prolonged in MMTV‐cNeu^tg/+^ SK4 KO mice (Fig. 4F). The important role of SK4 in this model was further confirmed *ex vivo* in MMTV‐cNeu^tg/+^‐derived breast cancer cells by demonstrating the TRAM‐34 sensitivity of their growth rate (Fig. S4A,B).

## 4 Discussion

Herein, we examined the role of endogenous SK4 channels for breast tumour cell growth, cell cycle progression, Ca^2+^ signalling and survival of breast tumour‐bearing PyMT and cNeu transgenic mice. In accordance with previous reports (Haren *et al*., 2010; Huang and Jan, 2014), we found high levels of SK4 expression in primary tumours of both animal models and in tumour cells derived thereof. Electrophysiological evidence for TRAM‐34‐sensitive, inwardly rectifying currents in SK4 WT but not SK4 KO PyMT oncogene‐driven breast tumour cells (Fig. 1) supports the notion that the SK4 channel is functional in breast tumour cells. Based on these initial experiments, tumour formation and development were evaluated in the absence of SK4 *in vivo*. Ablation of SK4, however, had a minor effect on mean survival rates but did not significantly prolong tumour‐free survival nor overall survival in the MMTV‐PyMT^tg/+^ model (Fig. 4), possibly due to an impaired immune and/or stromal cell response, which results in a tumour progression‐promoting microenvironment (Jinushi *et al*., 2011; Loeffler *et al*., 2006; Wiseman and Werb, 2002) that counteracts the loss of the oncogenic SK4 functions in the tumour cells. The difference in tumour formation time between SK4‐deficient and SK4‐proficient MMTV‐PyMT tumour cells transplanted orthotopically into WT mice strongly supports this hypothesis. Indeed, recent evidence from a melanoma‐bearing mouse model suggests that the potassium efflux via SK4 and Kv1.3 is essential for tumour‐specific T cells in order to impair malignant growth (Eil *et al*., 2016). The oncogenic role of SK4 was further confirmed by the MMTV‐cNeu^tg/+^ breast tumour model where SK4 ablation prolonged the survival rates. Unfortunately, the role of SK4 in different cells and compartments of the tumour microenvironment, that is in noncancerous cells such as tumour‐associated immune cells, endothelial cells and/or cancer‐associated fibroblasts (CAFs) (Koshy *et al*., 2013; Xu *et al*., 2014), could not be tested by the present study due to the lack of a conditional SK4 KO mouse line allowing for the generation of cell‐specific SK4 mutants.

Our observation that both growth factor‐evoked and spontaneous Ca^2+^ transients in PyMT breast tumour cells do require SK4 activity is consistent with the TRAM‐34 sensitivity of the cell cycle and abundance of *c‐fos* and *c‐jun* (Lai *et al*., 2011; Ouadid‐Ahidouch *et al*., 2004; Wang *et al*., 2007b). Previous work identified hERG1, Kv1.1, BK and several other K^+^ channels including SK4 to cause membrane hyperpolarization, which modifies the passive Ca^2+^ entry in nonexcitable cancer cells (Ouadid‐Ahidouch and Ahidouch, 2013). Although the molecular identity for the SK4‐controlled Ca^2+^ entry routes, that is the plasma membrane channels and store‐operated mechanisms in the PyMT and Her2/cNeu breast tumour cells, is ill‐defined, SK4 was previously colocalized or functionally linked to Ca^2+^ release‐activated Ca^2+^ (CRAC)‐like channels or TRP (transient receptor potential) family such as TRPV6, TRMP7 and TRMP3 in T lymphocytes, prostate cancer cells (Kuras *et al*., 2012; Lallet‐Daher *et al*., 2009) and MMTV‐PyMT^tg/+^ breast tumour cells (data not shown).

Our data point to an oncogenic role of SK4 in mice that may have implications for human breast cancer. In humans, overexpression is considered the main mechanism of oncochannels to contribute to the malignancy of cancer (Huber, 2013; Pardo and Stuhmer, 2014), but activating mutations in the SK4‐encoding *KCNN4* gene may also contribute to the expression of abnormally regulated channels. Notably, KCNN4 promoter hypomethylation is associated with high levels of SK4 in aggressively growing non‐small‐cell lung carcinoma cell lines and advanced stages of lung cancer in patients (Bulk *et al*., 2015). With regard to breast cancer, similar epigenetic changes may account for progression; however, such mechanisms have not been elucidated as of yet. Rather, at the germline level, several intronic KCNN4 single nucleotide polymorphisms (SNPs) have been shown to alter women's breast cancer risk in general and particularly of ER‐positive breast cancer. The observed effect size is small, but is of genome‐wide significance (*P* < 10^−6^) (Lo *et al*., 2016; Michailidou *et al*., 2015). How the genetic variants affect SK4 expression in normal breast tissue and breast tumour is currently unknown.

Together, our findings from animal models suggest that the role of SK4 channels should be further explored within the context of breast cancer progression and as a new strategy for future breast cancer management. It seems to be necessary to define the effect of such an approach on SK4‐dependent pathways in nontumour cells and in the context of the mutated oncogenes. This is important, as SK4 and SK4‐dependent pathways might hold the potential to establish novel breast cancer drug targets.

## Author contributions

FAS, CJM, PR and RL were involved in research design. FAS, CJM, BS, HYN, AB and MS conducted experiments. SBH and PK contributed new reagents, techniques or analytic tools. FAS, CJM, BS, MS, SMH and RL analysed the data. WYL, WS, RH and HB contributed to discussions. FAS, CJM, BS, WYL, WS, RH, HB, PR, SMH and RL wrote or contributed to the writing of the manuscript. All authors approved the final manuscript.

## Supporting information


**Fig. S1.** (A) Relative SK4 expression in malignant breast tumour tissue compared to healthy mammary gland tissue of female non‐transgenic FVB/N wildtype mice. Statistical analysis was performed using an unpaired student's *t*‐test (**P < 0.01). (B) SK4 expression in MMTV‐cNeu^tg/+^ breast tumour cells. mRNA isolated from SK4 WT and SK4 KO thymus was used to control the specificity of the primer pairs used. (C–D) Relative expression levels of SK1‐3 and SK4 mRNA in MMTV‐PyMT^tg/+^ WT breast tumour samples. (E) Apoptosis induction by TRAM‐34 was monitored by cytochrome c release of mitochondria into the cytosol. Cells were treated for 4, 24 and 48 h with TRAM‐34 (10 μM) and staurosporine 1 μM as positive control. Mitochondrial and cytosolic fraction were separated and analysed by SDS‐PAGE and Western blot. As expected, staurosporine induced cytochrome c release and cleavage of caspase‐3, whereas no corresponding signals were observed in the TRAM‐34 treated cells. HSP60 was used as mitochondrial marker and α‐Tubulin as a marker for the cytosolic fraction. Sufficient separation of mitochondrial and cytosolic fractions in this setup was proven by Western blot analysis of mitochondrial samples (data not shown).
**Fig. S2.** (A) SK4 WT MMTV‐PyMT^tg/+^ and (B) SK4 KO MMTV‐PyMT^tg/+^ breast tumour cells grown for 72 h in the absence and presence of 0.1, 1.0 or 10 μM TRAM‐34 (*n* = 5 independent experiments per genotype and treatment condition).
**Fig. S3.** Ki‐67 expression in MMTV‐PyMT^tg/+^ breast tumour cells (SK4 WT vs. SK4 KO).
**Fig. S4.** (A) Effect of TRAM‐34 (10 μM) or vehicle (CTR) on the growth of MMTV‐cNeu^tg/+^ SK4 WT cells. Representative pictures were acquired at the different time points indicated in the mini‐grid assay (scale bar = 100 μm). (B) Cells depicted in (A) were counted with imagej Software version 1.46 and cell numbers were normalized to t_0_ for each time point and treatment (*n* = 10). Statistical analysis was performed by one‐way ANOVA followed by Bonferroni correction (**P < 0.01).
