# Supplementary material for: SK4 channels modulate Ca2+ signalling and cell cycle progression in murine breast cancer
Source: Mol Oncol. 2017 Jun 26;11(9):1172–88. doi: 10.1002/1878-0261.12087 (PMC5579333; doi:10.1002/1878-0261.12087)
Supplement: Supplementary file 1 — Fig. S1. (A) Relative SK4 expression in malignant breast tumour tissue compared to healthy mammary gland tissue of female non‐transgenic FVB/N wildtype mice. Statistical analysis was performed using an unpaired student's t‐test (**P < 0.01). (B) SK4 expression in MMTV‐cNeutg/+ breast tumour cells. mRNA isolated from SK4 WT and SK4 KO thymus was used to control the specificity of the primer pairs used. (C–D) Relative expression levels of SK1‐3 and SK4 mRNA in MMTV‐PyMTtg/+ WT breast tumour samples. (E) Apoptosis induction by TRAM‐34 was monitored by cytochrome c release of mitochondria into the cytosol. Cells were treated for 4, 24 and 48 h with TRAM‐34 (10 μM) and staurosporine 1 μM as positive control. Mitochondrial and cytosolic fraction were separated and analysed by SDS‐PAGE and Western blot. As expected, staurosporine induced cytochrome c release and cleavage of caspase‐3, whereas no corresponding signals were observed in the TRAM‐34 treated cells. HSP60 was used as mitochondrial marker and α‐Tubulin as a marker for the cytosolic fraction. Sufficient separation of mitochondrial and cytosolic fractions in this setup was proven by Western blot analysis of mitochondrial samples (data not shown). Fig. S2. (A) SK4 WT MMTV‐PyMTtg/+ and (B) SK4 KO MMTV‐PyMTtg/+ breast tumour cells grown for 72 h in the absence and presence of 0.1, 1.0 or 10 μM TRAM‐34 (n = 5 independent experiments per genotype and treatment condition). Fig. S3. Ki‐67 expression in MMTV‐PyMTtg/+ breast tumour cells (SK4 WT vs. SK4 KO). Fig. S4. (A) Effect of TRAM‐34 (10 μM) or vehicle (CTR) on the growth of MMTV‐cNeutg/+ SK4 WT cells. Representative pictures were acquired at the different time points indicated in the mini‐grid assay (scale bar = 100 μm). (B) Cells depicted in (A) were counted with imagej Software version 1.46 and cell numbers were normalized to t0 for each time point and treatment (n = 10). Statistical analysis was performed by one‐way ANOVA followed by Bonferroni correction (**P < 0. [file MOL2-11-1172-s001.docx]

# SK4 channels modulate Ca^2+^-signalling and cell cycle progression in murine breast cancer

Friederike A. Steudel^1^, Corinna J. Mohr^1,2^, Benjamin Stegen^3^, Hoang Y Nguyen^1^, Andrea Barnert^1^, Marc Steinle^1^, Sandra Beer-Hammer^4^, Pierre Koch^5^, Wing-Yee Lo^2^, Werner Schroth^2^, Reiner Hoppe^2^, Hiltrud Brauch^2,6^, Peter Ruth^1^, Stephan M. Huber^3^, Robert Lukowski^1,§^

^1^Department of Pharmacology, Toxicology and Clinical Pharmacy, Institute of Pharmacy, University of Tuebingen, 72076 Tuebingen, Germany

^2^Dr. Margarete Fischer-Bosch - Institute of Clinical Pharmacology, 70376 Stuttgart and University of Tuebingen, 72074 Tuebingen, Germany

^3^Department of Radiation Oncology, University of Tuebingen, 72076 Tuebingen, Germany

^4^Department of Pharmacology and Experimental Therapy, Institute of Experimental and Clinical Pharmacology and Toxicology, University Hospital Tuebingen, 72074 Tuebingen, Germany

^5^Pharmaceutical and Medicinal Chemistry, Institute of Pharmacy, University of Tuebingen, 72076 Tuebingen, Germany

^6^German Cancer Consortium (DKTK), German Cancer Research Center (DKFZ), Heidelberg, Germany.

^§^**Corresponding author:** Robert Lukowski, Department of Pharmacology, Toxicology and Clinical Pharmacy, Institute of Pharmacy, University of Tuebingen, Germany Tel. +49 7071 29 74550, Fax +49 7071 29 2476, E-mail: robert.lukowski@uni-tuebingen.de

**Running Title:** SK4 in breast cancer cell growth and Ca^2+^-entry

**Supplemental information:** Supplemental Figures and Figure Legends (1-4)

**Supplemental Figures and Legends**


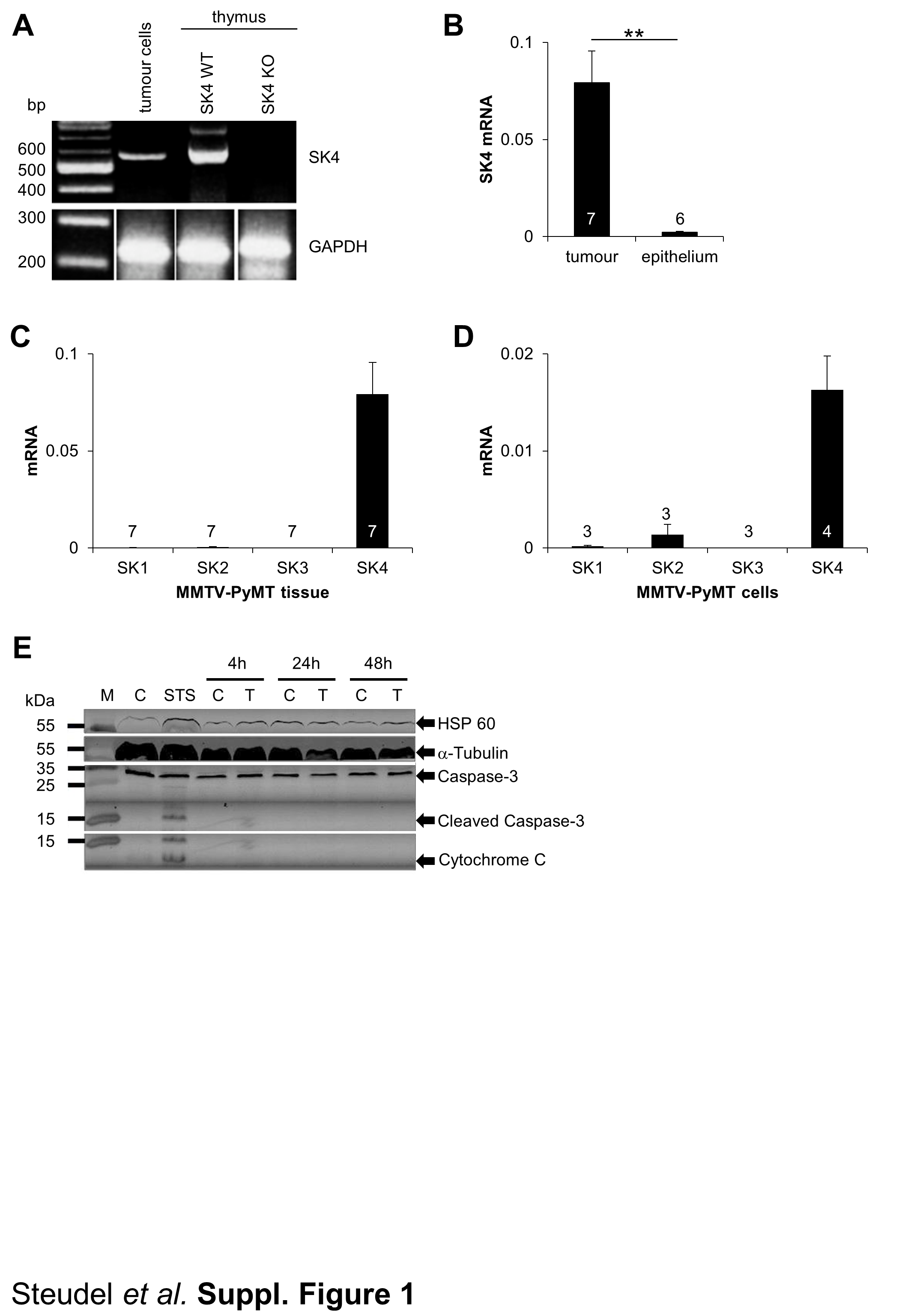


**Suppl. Fig. 1**

**(a)** Relative SK4 expression in malignant breast tumour tissue compared to healthy mammary gland tissue of female non-transgenic FVB/N wildtype mice. Statistical analysis was performed using an unpaired student’s t-test (** p<0.01).

**(b)** SK4 expression in MMTV-cNeu^tg/+^ breast tumour cells. mRNA isolated from SK4 WT and SK4 KO thymus was used to control the specificity of the primer pairs used.

**(c-d)** Relative expression levels of SK1-3 and SK4 mRNA in MMTV-PyMT^tg/+^ WT breast tumour samples.

**(e)** Apoptosis induction by TRAM-34 was monitored by cytochrome c release of mitochondria into the cytosol. Cells were treated for 4, 24 and 48 h with TRAM-34 (10 µM) and staurosporine 1 µM as positive control. Mitochondrial and cytosolic fraction were separated and analysed by SDS-PAGE and Western blot. As expected, staurosporine induced cytochrome c release and cleavage of caspase-3, whereas no corresponding signals were observed in the TRAM-34 treated cells. HSP60 was used as mitochondrial marker and α-Tubulin as a marker for the cytosolic fraction. Sufficient separation of mitochondrial and cytosolic fractions in this setup was proven by Western blot analysis of mitochondrial samples (data not shown).

Abbreviations used: *SK4*: Calcium-activated potassium channel with intermediate conductance; *bp*: base pairs; *WT*: wildtype; *KO*: knockout; *GAPDH*: Glyceraldehyde 3-phosphate dehydrogenase; *CTR*: control; *TRAM-34*: triarylmethan-34; *h*: hours; *kDa*: kilodalton; *M*: marker; *C*: control; *STS*: staurosporine; *T*: TRAM-34; *HSP60*: heat shock protein 60.

**
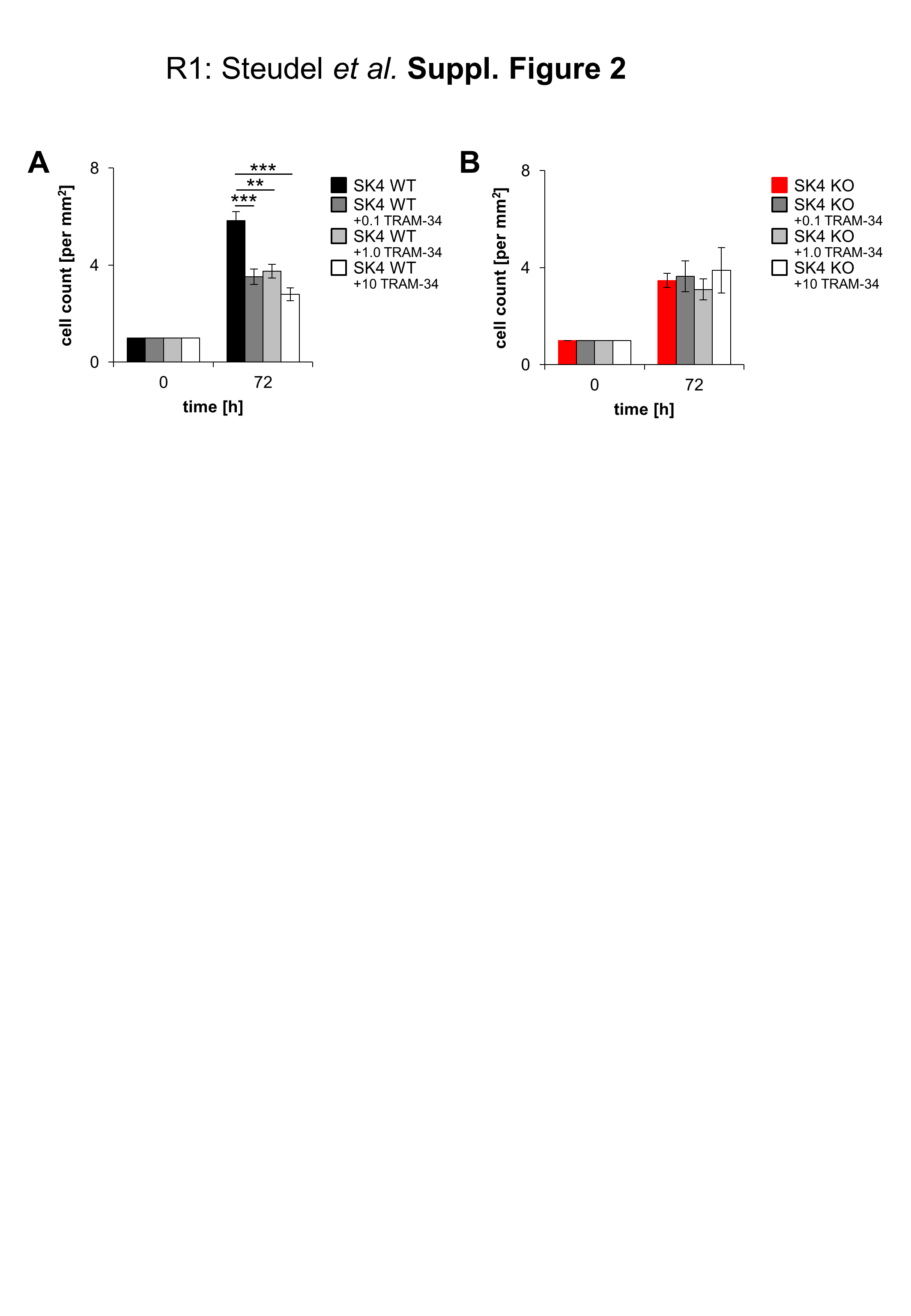
**

**Suppl. Fig. 2**

**(a)** SK4 WT MMTV-PyMT^tg/+^ and **(b)** SK4 KO MMTV-PyMT^tg/+^ breast tumour cells grown for 72 h in the absence and presence of 0.1, 1.0 or 10 µM TRAM-34 (n=5 independent experiments per genotype and treatment condition). Statistical analysis was performed by one-way ANOVA and Tukey´s multiple comparisons test (** p<0.01; *** p<0.001).

Abbreviations used: *SK4*: Calcium-activated potassium channel with intermediate conductance; *WT*: wildtype; *TRAM-34*: triarylmethan-34 *KO*: knockout; *h:* hours; *MMTV:* mouse mammary tumour virus; *PyMT:* polyoma virus middle T antigene.


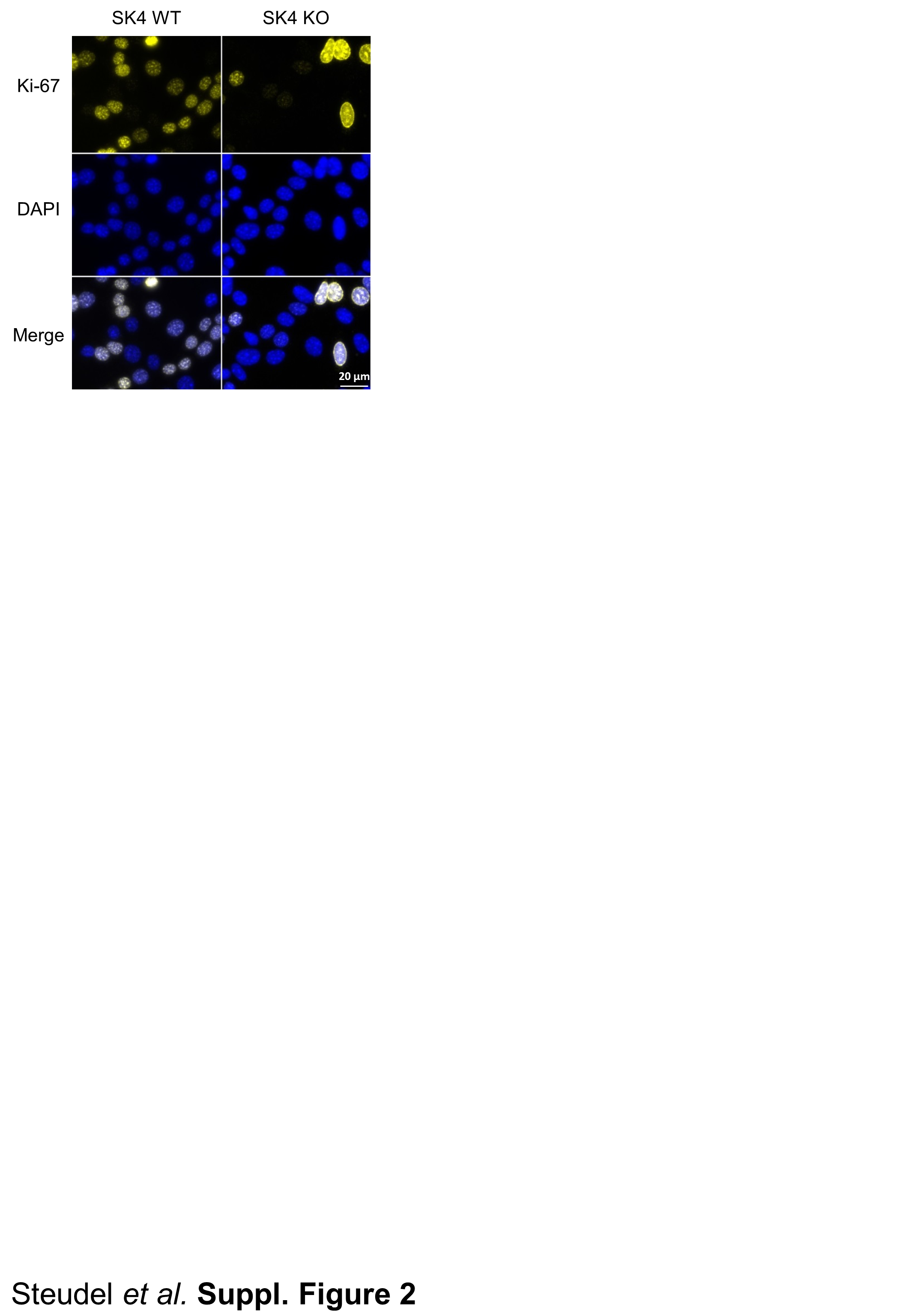


**Suppl. Fig 3**

Ki-67 expression in MMTV-PyMT^tg/+^ breast tumour cells (SK4 WT vs. SK4 KO). Cells were grown for 24, 48 or 72 h. The fraction of Ki-67-positive cells in each sample was obtained by assessing the number of Ki-67-positive cells in relation to the total number of nuclei stained with DAPI.

Abbreviations used: *SK4*: Calcium-activated potassium channel with intermediate conductance; *WT*: wildtype; *KO*: knockout; DAPI: 4,6-Diamidino-2-phenylindole, dihydrochloride


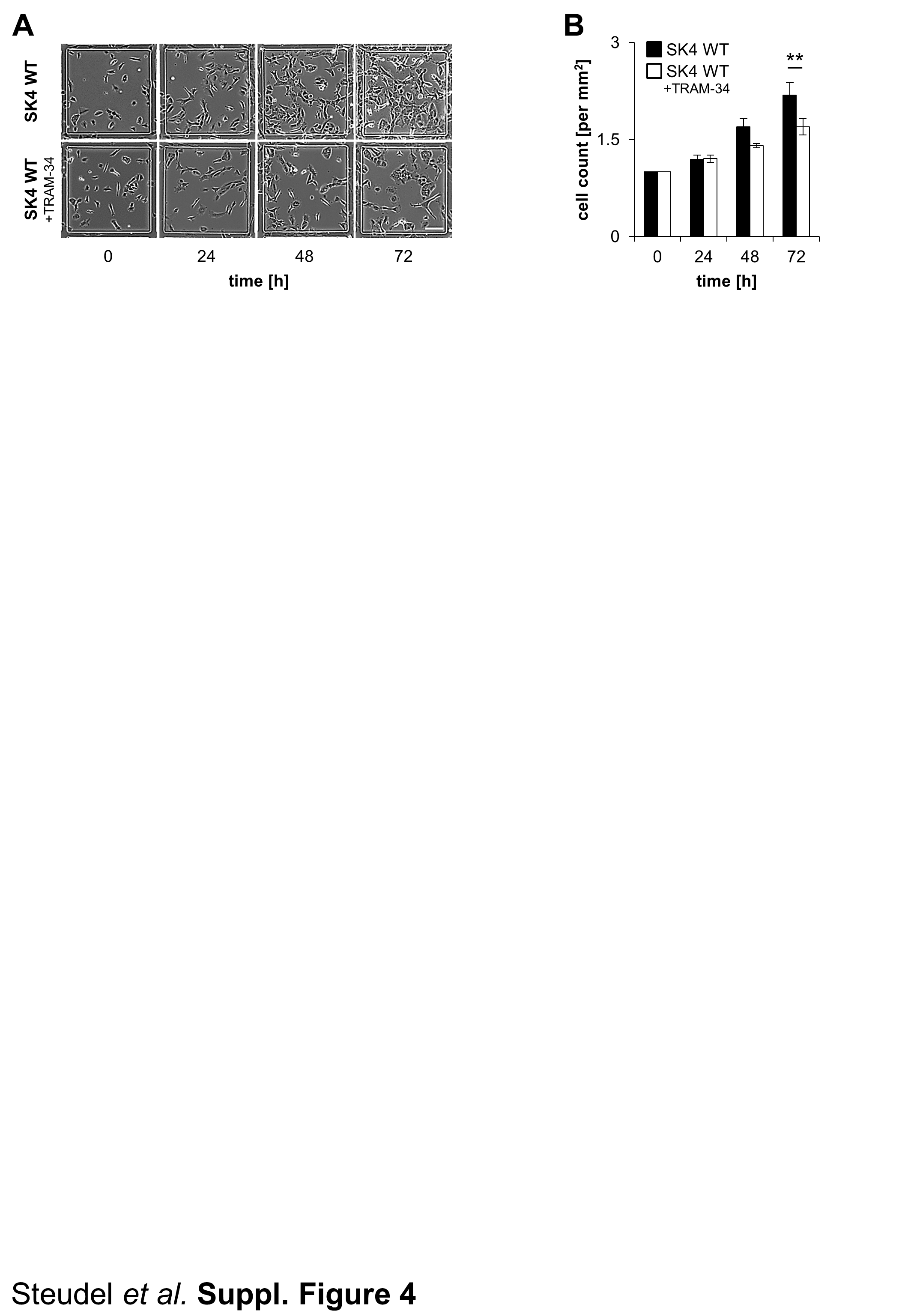


**Suppl. Fig 4**

**(a)** Effect of TRAM-34 (10 µM) or vehicle (CTR) on the growth of MMTV-cNeu^tg/+^ SK4 WT cells. Representative pictures were acquired at the different time points indicated in the mini-grid assay (scale bar = 100 µm).

**(b)** Cells depicted in (a) were counted with ImageJ Software version 1.46 and cell numbers were normalized to t0 for each time point and treatment (n=10). Statistical analysis was performed by one-way ANOVA followed by Bonferroni correction (** p<0.01).

Abbreviations used: *SK4*: Calcium-activated potassium channel with intermediate conductance; *WT*: wildtype; *TRAM-34*: triarylmethan-34; *h*: hours)
